# Quality in Use in Connected Mental Health: Protocol for a Systematic Mapping Study

**DOI:** 10.2196/79611

**Published:** 2026-01-20

**Authors:** Shweta Premanandan, Sofia Ouhbi, Magdalena Ramstedt Stadin, Charlotte Blease, Åsa Cajander, Maria Hägglund

**Affiliations:** 1 Division of Computing Science Department of Information Technology Uppsala University Uppsala Sweden; 2 Division of Vi3 Department of Information Technology Uppsala University Uppsala Sweden; 3 Participatory eHealth and Health Data Research Group Department of Women's and Children's Health Uppsala University Uppsala Sweden

**Keywords:** connected mental health, quality in use, systematic mapping study, digital mental health evaluation, user-centered design, eHealth, mental health technologies, usability, human-centered evaluation

## Abstract

**Background:**

Quality in use (QiU), a stakeholder-centered dimension of software quality encompassing effectiveness, efficiency, satisfaction, and freedom from risk, is essential in evaluating digital systems, particularly in health-related domains. Although QiU has been explored in various fields, its application within connected mental health (CMH) systems remains fragmented and understudied. Given the rapid rise in CMH technologies, ranging from mobile apps to teletherapy platforms, understanding how QiU is conceptualized, evaluated, and reported in this domain has become increasingly urgent.

**Objective:**

This study aims to systematically map and synthesize existing research on QiU in CMH applications. It seeks to identify current trends, research gaps, evaluation methods, and the range of technologies examined concerning QiU.

**Methods:**

A systematic mapping methodology following the guidelines by Petersen et al will be used. The process includes defining mapping questions, developing a classification scheme, and systematically searching and analyzing peer-reviewed literature from databases—Scopus, PubMed, IEEE Xplore, and ACM Digital Library. Eight mapping questions will guide the analysis, focusing on publication trends, research types, empirical evaluations, QiU characteristics and subcharacteristics, and technologies studied.

**Results:**

As this paper presents the protocol for an ongoing mapping study, results are not yet available. The literature search and data analysis are scheduled for completion in 2026. Preliminary screening suggests variability in how QiU is defined and evaluated across CMH technologies, highlighting the need for systematic synthesis.

**Conclusions:**

This systematic mapping study will fill a critical gap by providing a comprehensive overview of QiU research in the context of CMH. By organizing and classifying the existing literature, the study will inform future research, support the development of more user-centered CMH tools, and contribute to establishing more consistent evaluation practices in this growing field.

**International Registered Report Identifier (IRRID):**

DERR1-10.2196/79611

## Introduction

### Background

**Figure 1:**
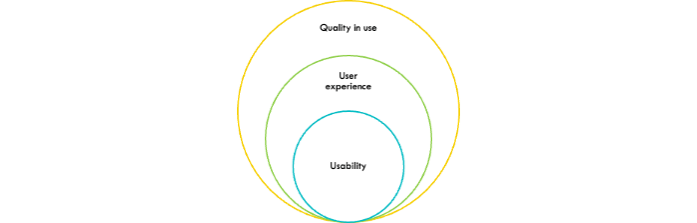
Relationship between usability, user experience, and quality in use.

Quality in use (QiU) is an important aspect of software evaluation, focusing on the stakeholders’ perspective and experience. The ISO/IEC 25019:2023 standard defines QiU as “extent to which the system or product when it is used in a specified context of use satisfies or exceeds stakeholders needs to achieve specified beneficial goals or outcomes” [1], which has replaced the ISO/IEC 25010:2011 standard, defines QiU as the “extent to which the system or product when it is used in a specified context of use satisfies or exceeds stakeholders needs to achieve specified beneficial goals or outcomes” [2]. Measuring QiU presents challenges due to complex standard models and limitations of customized quality models [3]. Researchers have proposed various approaches to assess QiU, including latent semantic analysis of user reviews [4], sentiment analysis [3], and systematic evaluation frameworks [5]. QiU models have been developed for specific applications such as web portals [6] and e-learning systems [5]. The concept of QiU extends beyond usability and user experience (UX; Figure 1), integrating human factors into the software engineering life cycle [2].

To establish a clear scope for this mapping study, we explicitly define QiU as outlined in the widely used quality framework in software engineering in the ISO/IEC 25010:2011 standard and its replacement in terms of QiU, the ISO/IEC 25019:2023 standard. Therefore, QiU goes beyond traditional constructs such as usability (focused primarily on effectiveness, efficiency, and satisfaction during interaction), UX (the broader set of emotional, cognitive, and contextual responses), and freedom from risk (minimizing potential harm). While these concepts overlap, QiU represents an integrative, outcome-oriented perspective that links interaction, performance, and user-perceived value in real contexts of use.

In this study, we will map QiU dimensions, methods used to evaluate QiU, and conceptual or analytical frameworks related to QiU in connected mental health (CMH). Pure usability studies or UX-focused evaluations will be included only when they explicitly address or report on one or more QiU characteristics. To support transparency and avoid conceptual drift, we have added a visual conceptual model (Figure 1) that illustrates how QiU relates to but remains distinct from usability, UX, and safety in CMH technologies. This model anchors the boundaries of what is being mapped and ensures clear differentiation from prior CMH usability reviews.

CMH has emerged as a crucial tool for addressing the global mental health crisis, offering innovative solutions to improve access to and quality of care [7,8]. These technologies range from smartphone apps and virtual reality to artificial intelligence–powered chatbots, providing support, prevention, and treatment options [7]. CMH has shown promise in improving mental health outcomes, such as in the case of underserved populations such as lesbian, gay, bisexual, transgender, and queer individuals [9]. However, challenges remain, including ensuring efficacy and user safety and addressing the digital divide [10]. Ethical considerations such as data privacy and the need for evidence-based practices are crucial [11]. Despite these challenges, investing in CMH presents significant economic and societal benefits, especially in low-resource settings [12]. As the field evolves, focus should be placed on meaningful community engagement, personalization, and integration with traditional health care models [7,13].

As CMH technologies become increasingly embedded in mental health care delivery, evaluating these tools from a user-centered perspective is crucial. Unlike traditional clinical interventions, CMH applications are often accessed independently by users without direct supervision from mental health professionals (eg, psychologists or psychiatrists). This autonomy underscores the importance of QiU as a critical determinant of the effectiveness, engagement, and long-term adoption of CMH applications. Evaluating QiU allows developers and researchers to better understand how users experience these technologies in real-world settings, including aspects such as trust, perceived safety, ease of use, and usefulness, factors that directly influence outcomes such as adherence and therapeutic benefit [14].

Moreover, CMH interventions must be particularly sensitive to user vulnerabilities, ethical risks, and contextual appropriateness. Poorly designed systems may not only fail to provide support but can also actively cause harm, for instance, by triggering distress or mishandling sensitive data [15]. Therefore, QiU is not just a technical consideration but an ethical imperative in the design and deployment of CMH technologies.

While the broader literature on CMH has grown rapidly [8], few reviews have specifically addressed how QiU is conceptualized, evaluated, or reported in the CMH domain. Existing systematic reviews have tended to focus on clinical effectiveness [16], usability testing [14], or specific populations and conditions [15], often treating UX as a secondary concern. Furthermore, evaluations often rely on ad hoc instruments rather than established standards such as the ISO/IEC 25010, leading to inconsistent reporting and a lack of comparability across studies [1].

There is also considerable variability in how QiU subcharacteristics are interpreted and applied in CMH research. For example, usability is frequently assessed, but other critical dimensions such as safety, learnability, and risk mitigation receive little attention [1]. Additionally, there is a lack of clarity on which types of technologies, user groups, and mental health contexts have been studied in relation to QiU. Without a comprehensive overview of these patterns, it remains difficult to build a coherent body of knowledge to inform both theory and practice.

This systematic mapping study addresses these gaps by organizing and analyzing the existing literature on QiU in CMH applications. By identifying trends, challenges, and underexplored areas, it aims to support more consistent, rigorous, and user-centered evaluation practices in CMH.

### Study Objectives

This systematic mapping study aims to identify, categorize, and synthesize research evidence on how QiU is evaluated in CMH applications with the purpose of informing more robust, user-centered design and evaluation practices.

## Methods

### Overview

This study will adopt the mapping process outlined by Petersen et al [17], which involves selecting relevant publications, developing a classification scheme, and systematically mapping the literature. The primary goal of a systematic mapping study is to organize a research field and provide a comprehensive overview of existing literature, focusing on exploring topics and categorizing the available contributions [18].

Our research team brings together international and interdisciplinary perspectives from medical informatics, human-computer interaction, information systems, and software engineering and includes researchers at different career stages (from early-career researchers to professors). This heterogeneity provides a broad understanding of digital health, sociotechnical systems, user-centered evaluation, and software quality, enriching the analysis and reducing the dominance of any single disciplinary lens. All coders have prior experience conducting systematic literature reviews [19,20], scoping reviews [21,22], or mapping studies [8,23], and several have expertise in usability testing, digital health evaluation, participatory design, or software quality.

We acknowledge that our positionalities as academic researchers working within digital health, sociotechnical, and design-oriented communities may influence how we interpret constructs related to QiU, such as usability, satisfaction, trust, or freedom from risk. While we do not have clinical roles or direct relationships with the study populations, our collective orientation toward user-centered and standard-driven evaluation may predispose us to notice certain QiU subcharacteristics more readily than others. To mitigate these potential influences, we anchor our classification scheme in the ISO/IEC 25019:2023 and ISO/IEC 25010:2011 models and use systematic intercoder reliability procedures: 2 independent reviewers will screen and code each study, discrepancies will be resolved through consensus, and a senior member will review unresolved conflicts.

### Mapping Questions

Textbox 1 presents the 8 mapping questions. The mapping questions were defined to provide an overview and a structured understanding of the existing literature on QiU in CMH.

**Mapping questions.**

- What is the temporal distribution of publications in this field? (Mapping question 1)
- What are the publication avenues of quality in use (QiU) in connected mental health (CMH) research? (Mapping question 2)
- What are the research types in which QiU is used in CMH? (Mapping question 3)
- What types of mental health problems are addressed in the QiU literature? (Mapping question 4)
- What are the types of empirical evaluation, if any (eg, case studies or interviews), of QiU in CMH research? (Mapping question 5)
- What QiU characteristics or subcharacteristics are reported in the CMH literature? (Mapping question 6)
- What are the key challenges or limitations identified in QiU in CMH? (Mapping question 7)
- What technologies (eg, mobile apps, wearable devices, or telehealth) are studied for QiU in CMH? (Mapping question 8)

### Search Strategy

The review will analyze studies that discuss QiU in CMH applications. The search strategy for this systematic mapping study was developed in collaboration with Görel Sundström, a librarian from Uppsala University, and the research team (SP, SO, MH, CB, ÅC, and MRS). The population, intervention, comparator, and outcome framework was used to construct the search strategy, as shown in Table 1.

**Table 1:** Preliminary search strategy.

Search number	Keyword	Database search algorithm
1	“Quality in use”	TITLE-ABS-KEY (“IEC 25010” OR “ISO 25010” OR “ISO/IEC 25010” OR “ISO 25019” OR “IEC 25019” OR “ISO/IEC 25019” OR “quality-in-use” OR “quality in use” OR accessib* OR appropriateness OR beneficialness OR effectiveness OR efficiency OR flexibility OR “freedom from risk” OR “interaction capability” OR “interface design” OR learnability OR operability OR quality OR recognizability OR “risk mitigation” OR safe* OR satisfaction OR suitab* OR trust* OR usability OR usefulness OR “user experience*” OR UX OR “user need*”)
2	“Connected mental health”	TITLE-ABS-KEY (“e-mental health” OR “m-mental health” OR ((“mental health” OR psychotherapy OR “behavi* therapy”) W/2 (connected OR digital OR mobile OR online OR smart OR tele OR video OR web)))
3	“Systems”	TITLE-ABS-KEY (app OR apps OR application* OR software OR system OR systems OR device*)
4	—^a^	Search 1 AND search 2 AND search 3

^a^Not applicable.

Scopus was selected as the first database for this research due to its extensive collection of multidisciplinary academic content, covering fields such as engineering, medicine, business, and computer science [24]. The selection process aimed to identify the articles most relevant to this mapping study’s objectives.

The literature search will be conducted using multiple electronic databases: PubMed, IEEE Xplore, Scopus, and ACM Digital Library. These databases have been selected based on their relevance to the research topic and their widespread use in academic and scientific research. This systematic literature review will not involve the collection of any sensitive personal data.

### Study Selection Criteria

#### Overview

To ensure broad inclusion consistent with the goals of a mapping study, we defined the eligibility criteria based on the characteristics of the studies rather than on a clinical evidence framework. The criteria reflect the types of technologies, participants, evaluation focuses, and publication formats relevant to understanding QiU in CMH. This is detailed in Textbox 2.

**Criteria for inclusion and exclusion.**

**Inclusion criteria**

- Study focus or participants: studies involving end users (eg, patients, clinicians, or caregivers), stakeholders, or evaluators (eg, researchers or designers) who interact with or assess connected mental health (CMH) technologies; no restrictions on demographics or setting
- Technology or system type: information and communications technology tools explicitly related to CMH, including mobile apps, teletherapy platforms, chatbots, wearable-supported mental health interventions, and web-based self-help systems
- Evaluation focus: studies that report on aspects of quality in use (QiU; eg, usability, effectiveness, efficiency, safety, satisfaction, trust, or freedom from risk) in CMH applications, whether measured formally or described qualitatively
- Study type or methodology: qualitative methods, quantitative methods, mixed methods, solution proposals, evaluation research, and experience papers
- Publication type: formally published peer-reviewed journal articles and conference papers

**Exclusion criteria**

- Study focus or participants: studies not involving any users, stakeholders, or evaluators of CMH applications (eg, theoretical papers or technology descriptions without user focus)
- Technology or system type: interventions unrelated to mental health (eg, general wellness or physical health monitoring) or papers that discuss CMH without evaluating QiU
- Evaluation focus: studies that do not report on any user experience or QiU-related outcome or that focus solely on technical performance or clinical efficacy without user-centered evaluation
- Study type or methodology: reviews (eg, systematic reviews, scoping reviews, or meta-analyses)
- Publication type: gray literature, opinion pieces, protocols, and reviews

#### Types of Studies

##### Qualitative Studies

These encompass studies using methods such as interviews, focus groups, case studies, usability evaluations, participatory design, thematic analysis, grounded theory, and ethnographic observation that explore user or evaluator perspectives on CMH technologies.

##### Quantitative Studies

These encompass studies using experimental designs, surveys, user testing, task performance metrics, or statistical analysis to assess QiU aspects such as usability, efficiency, satisfaction, or effectiveness in CMH contexts.

##### Mixed Methods Studies

These encompass studies combining qualitative and quantitative approaches in a single research design to investigate user-centered evaluation of CMH applications. Examples include convergent, explanatory, or exploratory designs.

#### Language

Due to resource limitations, only studies published in English will be considered. The research team acknowledges that this may limit the inclusion of relevant studies published in other languages. This may introduce geographic and cultural bias, potentially limiting the diversity of perspectives and contexts represented in the findings.

#### Time Frame

No publication date limits will be applied to capture the full scope of research addressing QiU in CMH technologies.

### Study Screening

Librarian Görel Sundström initiated the search process using a set of predefined keywords that reflect the study’s inclusion and exclusion criteria. Duplicate records were removed using EndNote (version 21; Clarivate Analytics) according to the procedures outlined by Bramer et al [25]. The deduplicated results were then uploaded to Rayyan (Qatar Computing Research Institute) [26] for further processing. Screening will be conducted in 2 stages: first by evaluating titles and abstracts followed by full-text assessment. During the initial phase, at least 2 reviewers will independently screen the titles and abstracts, remaining blinded to each other’s decisions [27]. Any discrepancies during the full-text review will be resolved through discussion, and a third, independent reviewer will be involved when consensus cannot be reached.

### Data Extraction

The extraction of data from the selected studies will be guided by the mapping questions defined in this study. A standardized data extraction form will be developed collaboratively by the research team to ensure consistency. The extracted information will be used to categorize and classify the literature following the classification criteria derived from the mapping questions.

Specifically, data extraction will focus on the following dimensions:

- Bibliographic information (mapping questions 1 and 2)—includes author names, year of publication (temporal distribution), publication type (journal or conference), and country of study.
- Research characteristics (mapping questions 3 and 5)—captures the research type (qualitative, quantitative, or mixed methods), contribution type, and empirical method used (eg, usability study, interviews, surveys, or case studies).
- Technology and context (mapping questions 4 and 8)—documents the type of CMH technology studied (eg, mobile app, wearable, or telehealth platform), the mental health condition addressed (eg, anxiety, depression, or general well-being), and the user group (eg, patients, clinicians, or caregivers).
- QiU dimensions (mapping question 6)—extracts the subcharacteristics of QiU reported (eg, usability, satisfaction, effectiveness, or safety), along with any frameworks or tools used to assess them.
- Reported challenges (mapping question 7)—identifies limitations, risks, and barriers related to the evaluation or achievement of QiU in CMH systems.

All selected studies will be reviewed in full text to extract the required data. Two reviewers will carry out the initial data extraction, and a third reviewer will verify the results for accuracy and completeness. To ensure consistency between reviewers, we will assess interrater agreement using the Cohen κ coefficient [28] during the evaluation phase, along with the blind review feature in Rayyan, which provides an agreement percentage. Any discrepancies will be discussed with the third reviewer until consensus is reached. The extracted data will then be entered into a shared Microsoft Excel sheet for organization and analysis.

### Quality Assessment

Although formal quality appraisal is not always required in mapping studies, QiU research varies substantially in methodological rigor, from controlled laboratory usability tests to real-world clinical or field evaluations. To contextualize these differences, we will include a descriptive methodological quality coding for all included studies. This coding is based on the study by Ouhbi et al [29] and will capture key methodological attributes such as study design, evaluation setting, participant characteristics, data collection methods, and reported limitations.

### Data Analysis and Synthesis

Data from the included studies will be analyzed using quantitative and qualitative approaches guided by the 8 predefined mapping questions. Descriptive statistics will be used to address mapping questions 1 to 5 and 8, focusing on the temporal distribution of publications (mapping question 1), publication venues (mapping question 2), research types (mapping question 3), types of mental health and CMH technologies (mapping question 4), empirical evaluation methods (mapping question 5), and the technologies studied (mapping question 8). These patterns will be visualized through tables and charts generated in Microsoft Excel.

To address mapping questions 6 and 7, which relate to the reporting of QiU subcharacteristics and the challenges identified in CMH research, qualitative data will be analyzed thematically using Microsoft Excel. At least 2 reviewers will independently conduct the initial coding, and a third reviewer will independently review the data to ensure consistency.

Findings will be synthesized narratively combining quantitative trends with qualitative insights. Visual representations will be used to enhance clarity and support interpretation. The PRISMA (Preferred Reporting Items for Systematic Reviews and Meta-Analyses) 2020 flow diagram will be used to document and present the study selection process.

### Dissemination Strategy

The findings of this study will be published in a peer-reviewed scientific journal and presented at relevant academic conferences. In addition, the results will be shared through blog posts hosted by research groups affiliated with the authors.

### Ethical Considerations

According to the Ethical Review Act (2003:460) established by the Swedish Ethical Review Authority, this research is not subject to ethics approval requirements.

## Results

**Figure 2:**
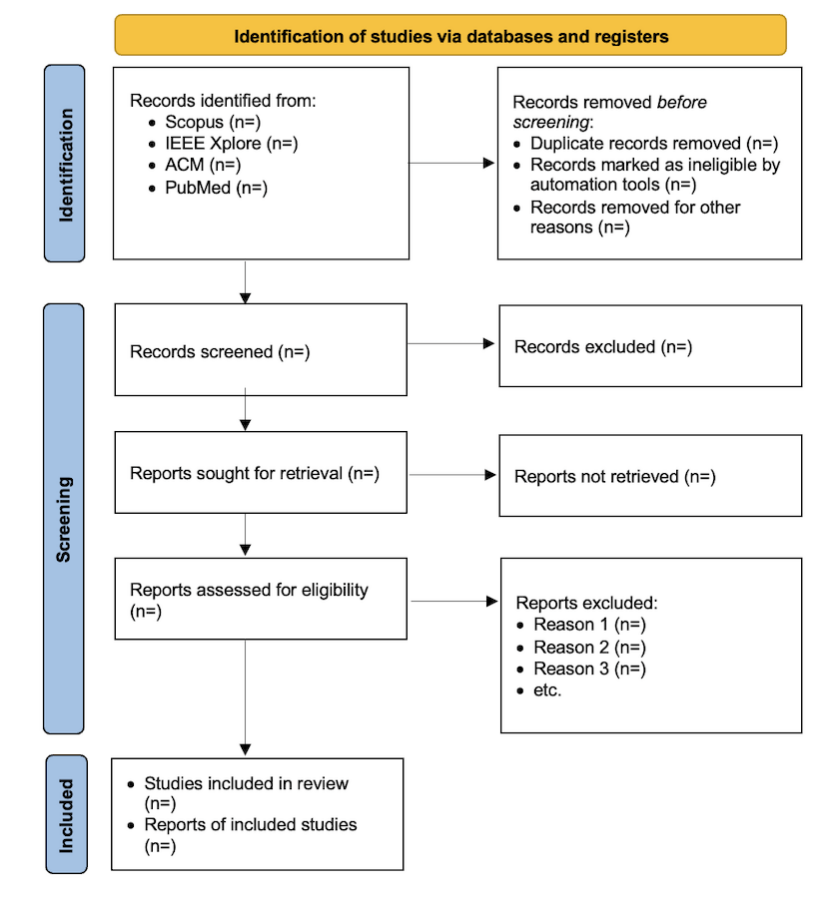
PRISMA (Preferred Reporting Items for Systematic Reviews and Meta-Analyses) flow diagram.

The study selection procedure will be illustrated using the PRISMA 2020 flow diagram [30], as demonstrated in Figure 2. The authors have so far conducted 2 pilot searches in collaboration with a librarian to refine the search terms and assess the initial relevance of the results. The outcomes are intended to be reported in a systematic mapping study and submitted for publication in 2026.

## Discussion

### Anticipated Principal Findings

On the basis of the scope of this mapping study, we anticipate 3 overarching patterns in how QiU is currently addressed in CMH research. First, QiU evaluations are expected to be highly heterogeneous, with studies variably emphasizing usability, UX, or safety rather than the integrated, standard-based definition provided by the ISO/IEC 25019:2023. Second, we expect to find a dominance of usability-focused assessments (eg, task success and satisfaction), with comparatively fewer studies addressing broader QiU dimensions such as context-of-use coverage, freedom from risk, or sustained goal achievement in real-world settings. Third, methodological diversity, ranging from laboratory usability tests to field deployments, is likely to reveal a fragmented evaluation landscape, suggesting the absence of a unified framework for QiU in CMH technologies. Together, these patterns will highlight both what the field currently measures and where systematic gaps limit our understanding of user-centered quality in mental health technologies.

### Comparison to Prior Work

Prior reviews in CMH have primarily focused on clinical effectiveness, usability, feasibility, or ethical considerations rather than on a comprehensive assessment of QiU. For example, usability-oriented reviews highlight methodological inconsistencies, short-term evaluations, and limited attention to real-world contexts [14,16]. Broader CMH reviews have mapped technology types, use cases, and research trends [8,16], whereas others emphasize challenges related to privacy, safety, and the ethical implications of digital mental health tools [11,15]. Although these studies provide valuable insights, they typically address individual QiU-related constructs such as usability, acceptability, engagement, trust, or safety in isolation. None adopt an integrated, standard-based conceptualization anchored in the ISO/IEC 25010 or ISO/IEC 25019 standard, and few explicitly analyze how QiU subcharacteristics are explored or assessed in CMH research.

In contrast, the software engineering literature offers conceptual and methodological foundations for QiU, including theoretical models [2], application-specific frameworks [5,6], and computational approaches based on user-generated data [3,4]. However, these works rarely intersect with CMH and have not been synthesized in a mental health context. As a result, existing CMH evaluations often rely on ad hoc measures or usability-centric assessments rather than standardized QiU frameworks, contributing to fragmented reporting and limited comparability across studies [14,15]. Therefore, this mapping study is distinct from prior work as it is the first to integrate CMH and software engineering perspectives by systematically examining how QiU is conceptualized, operationalized, and evaluated in CMH technologies using the ISO/IEC 25019:2023 standard as an explicit conceptual anchor.

### Strengths and Limitations

A major strength of this mapping study is the adoption of a clear conceptual boundary for QiU based on the ISO/IEC 25019:2023 and ISO/IEC 25010:2011 standards, which reduces the ambiguity that could limit comparability across CMH evaluations. Additionally, the eligibility framework was intentionally designed to be broad, enabling the inclusion of design studies, exploratory investigations, mixed methods evaluations, and experience reports from diverse mental health contexts.

However, some limitations are anticipated. First, QiU terminology is inconsistently used across the CMH literature, which may result in implicit QiU evaluations being categorized under related constructs (eg, usability or acceptability). However, this risk is mitigated because the search string includes terms related to QiU. Second, because QiU encompasses broad real-world outcomes, studies using short-term or laboratory-based assessments may contribute unevenly to the mapping. Third, like any mapping study, the methodological approach based on the established framework by Petersen et al [17] represents one of several possible ways to structure and categorize a broad field. Alternative strategies for data gathering could yield different groupings or emphases, and therefore, our findings should be interpreted as one evidence-based representation among multiple possible perspectives. Fourth, the study focuses exclusively on peer-reviewed journals and conference publications. Although this approach enhances methodological rigor and transparency, it excludes gray literature, dissertations, practitioner reports, and informal sources that may contain emerging ideas or practical insights. Fifth, while we use multiple independent reviewers during screening, coding, and verification to minimize individual bias, the interpretive nature of qualitative synthesis means that researcher perspectives may still influence decisions. We acknowledge that factors such as researcher background, expertise, and disciplinary orientation can shape categorization, and we have attempted to mitigate this through independent coding, verification by a third reviewer, and transparent reporting. Finally, the scope of this study is necessarily bounded by practical considerations, including available time, funding, and methodological feasibility. Broader analyses, such as the use of artificial intelligence–assisted synthesis techniques, the integration of sociocultural or political dimensions, multilingual considerations, or deeper engagement with stakeholder and developer perspectives, fall outside the remit of this protocol but represent promising areas for future research. By making these boundaries explicit, we aim to enhance the transparency of our approach and encourage subsequent studies to build on and extend the insights generated by this mapping study.

### Future Directions

The anticipated findings of this mapping study point to several promising directions for advancing QiU research in CMH. First, future studies would benefit from real-world, longitudinal evaluation designs that capture sustained UX, safety, and effectiveness beyond short-term laboratory interactions. Second, the adoption of standardized reporting guidelines is essential to enhance reproducibility and enable meaningful cross-study comparisons. Third, expanding QiU research to underrepresented populations and contexts, such as caregivers, adolescents, culturally diverse groups, and low-resource environments, could provide a more inclusive evidence base. Relatedly, expanding searches to include gray literature could strengthen and complement the peer-reviewed evidence base. Fourth, closer integration between design and evaluation processes, particularly by embedding QiU principles early in the development life cycle, can help ensure that CMH technologies align with users’ needs, risks, and real-world contexts. Overall, by illuminating how QiU is currently conceptualized and assessed, this mapping study establishes a foundation for more rigorous, comprehensive, and contextually grounded evaluation practices in CMH research.

### Conclusions

This mapping study will provide the first structured overview of how QiU has been conceptualized, operationalized, and evaluated in CMH technologies. The contribution of this work lies in establishing the conceptual boundaries of QiU, identifying the diversity of evaluation practices, and revealing where the field lacks coherence or methodological grounding. By synthesizing QiU dimensions, methods, and frameworks, the study will clarify how current research captures user-centered quality and where critical gaps remain, particularly in areas such as freedom from risk, context-of-use coverage, and real-world evaluation. The findings will support researchers and designers in selecting or developing QiU-aligned evaluation strategies and will help the CMH community move toward more consistent, standard-informed assessment practices. Ultimately, the mapping will serve as a foundation for future empirical work, methodological advancement, and the refinement of QiU models tailored to mental health technologies.

## Abbreviations

CMH: connected mental health
IEC: International Electrotechnical Commission
ISO: International Organization for Standardization
PRISMA: Preferred Reporting Items for Systematic Reviews and Meta-Analyses
QiU: quality in use
UX: user experience

## Data Availability

Data sharing is not applicable to this article as no datasets were generated or analyzed during this study.
